# Cryptorchidism and puberty

**DOI:** 10.3389/fendo.2024.1347435

**Published:** 2024-03-12

**Authors:** Wiwat Rodprasert, Helena E. Virtanen, Jorma Toppari

**Affiliations:** ^1^ Research Centre for Integrative Physiology and Pharmacology and Centre for Population Health Research, Institute of Biomedicine, University of Turku, Turku, Finland; ^2^ Department of Pediatrics, Turku University Hospital, Turku, Finland; ^3^ Department of Growth and Reproduction, Copenhagen University Hospital—Rigshospitalet, Copenhagen, Denmark; ^4^ Centre for Research and Research Training in Endocrine Disruption of Male Reproduction and Child Health (EDMaRC), Copenhagen University Hospital—Rigshospitalet, Copenhagen, Denmark

**Keywords:** undescended testis, Leydig cell, Sertoli cell, germ cell, testicular size, testosterone

## Abstract

Cryptorchidism is the condition in which one or both testes have not descended adequately into the scrotum. The congenital form of cryptorchidism is one of the most prevalent urogenital anomalies in male newborns. In the acquired form of cryptorchidism, the testis that was previously descended normally is no longer located in the scrotum. Cryptorchidism is associated with an increased risk of infertility and testicular germ cell tumors. However, data on pubertal progression are less well-established because of the limited number of studies. Here, we aim to review the currently available data on pubertal development in boys with a history of non-syndromic cryptorchidism—both congenital and acquired cryptorchidism. The review is focused on the timing of puberty, physical changes, testicular growth, and endocrine development during puberty. The available evidence demonstrated that the timing of the onset of puberty in boys with a history of congenital cryptorchidism does not differ from that of non-cryptorchid boys. Hypothalamic–pituitary–gonadal hormone measurements showed an impaired function or fewer Sertoli cells and/or germ cells among boys with a history of cryptorchidism, particularly with a history of bilateral cryptorchidism treated with orchiopexy. Leydig cell function is generally not affected in boys with a history of cryptorchidism. Data on pubertal development among boys with acquired cryptorchidism are lacking; therefore, more research is needed to investigate pubertal progression among such boys.

## Introduction

Uni- or bilateral cryptorchidism (undescended testis and maldescended testis) is the condition in which one or both testes have not descended adequately into the scrotum, respectively (1). Instead, the undescended testis is located anywhere along the normal path of testicular descent, which can be inside the abdominal cavity or in the inguinal canal, the suprascrotal position, or high scrotal areas (2). This condition is different from an ectopic testis, where the testis is located outside the normal path of testicular descent, for instance, in the femoral, perineal, or pubic area (3).

The testis originates in the abdominal cavity and is held by two structures—the suspensory ligament, attaching the upper part of the testis to the diaphragm, and the gubernaculum, anchoring the testis to the future inguinal area (4–7). Testicular descent is a complex process, and it is often described as two separate phases—the transabdominal phase and the inguinoscrotal phase (8). In the first phase, which occurs between the 10th and 15th gestational weeks, the testis migrates from the abdominal region to the area near the deep inguinal ring. The key hormone that drives this phase is insulin-like peptide 3 (INSL3) produced by Leydig cells. INSL3, by binding to its receptor [(relaxin-family peptide receptor-2 (RXFP2)] in the gubernaculum, causes shortening of the gubernacular cord and gubernacular bulb swelling, which keeps the testis close to the inguinal area during the fetal longitudinal growth. INSL3 also causes the widening of the inguinal canal for subsequent testicular descent (4–6). In the inguinoscrotal phase, occurring between the 25th and 35th gestational weeks, the testis moves from the inguinal region (through the inguinal canal) to the bottom of the scrotum—the process is driven by androgens (5, 6). It has been suggested that intra-abdominal pressure and maybe also the propulsive effect of muscles developed from the gubernaculum induce testicular descent into the scrotum (6, 9). Disruptions in the action of INSL3 or androgens (e.g., by environmental factors) or abdominal wall development can be associated with congenital cryptorchidism (10).

Congenital cryptorchidism is one of the most common urogenital abnormalities in male newborns, with a prevalence of 1.6%–9.0% at birth and 0.9%–1.8% at 3 months of age (6). The condition can be classified according to the possibly associated manifestations into syndromic and non-syndromic forms of congenital cryptorchidism. Syndromic cryptorchidism means that cryptorchidism is associated with a syndrome; i.e., there are also other clinical features than cryptorchidism. Examples of syndromic cryptorchidism are cryptorchidism associated with androgen insensitivity syndrome, sex chromosome abnormalities, and Noonan syndrome. However, most of the cryptorchid cases are non-syndromic (isolated), which means that cryptorchidism is not associated with other conditions (11).

Acquired cryptorchidism is the condition in which both testes descend adequately into the scrotum at birth, but one or both testes later ascend away from the normal position (12). Its prevalence varies between studies, at 0.6%–7% between the ages of 18 and 36 months and 1.1%–2.2% between the ages of 6 and 13 years (13–15). The causes, natural history, and management of this type of cryptorchidism are still unclear. The long-term health impact has been reported to be similar to that of the congenital form of cryptorchidism (16); therefore, it needs appropriate action.

Cryptorchidism is associated with an increased risk of infertility and a 4.8-fold increased risk of testicular germ cell tumors (17). However, the long-term sequelae of cryptorchidism on testicular hormone production, particularly during puberty, are not fully understood. Syndromic cryptorchidism, for instance, congenital hypogonadotropic hypogonadism, and deficiencies of enzymes in the androgen synthesis are frequently associated with androgen deficiency and pubertal delay or pubertal arrest (11). In contrast, data on testosterone levels and pubertal development in non-syndromic cryptorchidism are much less well-defined.

Orchiopexy is a surgical procedure to bring the undescended testis down to the proper position in the scrotum. This procedure includes different surgical techniques. For review, see Pakkasjärvi and Taskinen in *Frontiers in Endocrinology* 2024.

Here, we aim to review the current data on pubertal development only in boys with a history of non-syndromic cryptorchidism. Most currently available data are on congenital cryptorchidism, while data on acquired cryptorchidism are much more limited. First, the normal physiology of male pubertal development is briefly described. This is followed by pubertal development in boys with a history of congenital cryptorchidism, including the timing of puberty, physical changes, testicular growth, and endocrine development. Methods of testicular size measurement during puberty are briefly discussed. Reproductive hormone levels during minipuberty and puberty in boys with a history of congenital cryptorchidism are reviewed and discussed to show the endocrine changes during these two active periods of the hypothalamic–pituitary–gonadal axis. Testicular histopathology of congenital cryptorchidism is also reviewed shortly. Finally, pubertal development in acquired cryptorchidism is reviewed. The etiology and mechanisms of cryptorchidism are beyond the scope of this review.

## Physiology of male puberty

Puberty is the developmental period when the reproductive capacity develops. It is a transition period from childhood to adulthood, and it is associated with physical and mental changes. The increased activity of the hypothalamic–pituitary–gonadal (HPG) axis during puberty results in increased testicular testosterone production and the onset of testicular sperm production.

The activation of the HPG axis occurs in three phases of life—the fetal period, minipuberty, and puberty throughout adulthood. During minipuberty, serum follicle-stimulating hormone (FSH), luteinizing hormone (LH), testosterone, inhibin B, INSL3, and anti-Müllerian hormone (AMH) levels are elevated (18, 19). The high testosterone level in this period is associated with penile growth. FSH, LH, and testosterone levels decline toward 6 months of life; subsequently, LH and testosterone reach undetectable levels. The inhibin B level slowly declines after 3 months of age, reaching a low, but detectable level, in childhood after 1 year of age (18, 20–23). The AMH level increases during minipuberty and peaks at approximately 1–2 years of age; subsequently, the level slightly decreases to the prepubertal level. During minipuberty, gonocytes transform into A dark (Ad) spermatogonia, which are the adult stem cells for spermatogenesis (24). Sertoli cells proliferate, resulting in a slight testicular enlargement (25).

Inhibin B consists of α- and β-subunits. In childhood, both subunits are produced solely by Sertoli cells; thus, inhibin B level indicates Sertoli cell function and number. When meiosis starts during puberty, the α-subunits are produced by Sertoli cells, and β-subunits are produced by primary spermatocytes or early spermatids (23, 26). Therefore, inhibin B levels reflect the number and coordinated function of Sertoli cells and germ cells. Inhibin B suppresses FSH secretion from the anterior pituitary by a negative feedback control (26). In late puberty and adulthood, low inhibin B and high FSH levels indicate decompensated, reduced Sertoli cell and/or germ cell function or number, while normal inhibin B and elevated FSH levels indicate pituitary compensation for reduced Sertoli cell and/or germ cell function or number.

Puberty is initiated by the enhanced pulsatile gonadotropin-releasing hormone (GnRH) release from GnRH neurons in the hypothalamus, which stimulates the anterior pituitary to secrete FSH and LH. FSH stimulates Sertoli cells to support spermatogenesis and LH stimulates Leydig cells to produce testosterone, which is essential for the masculinization of boys, the maintenance of male characteristics in adulthood, and the support of spermatogenesis (27, 28). The function of GnRH neurons is regulated by several factors, one of which is the kisspeptin–GPR54 system. Kisspeptin, a peptide encoded by the *KISS1* gene, binds to its receptor, KISS1R, previously called G protein-coupled receptor 54 (GPR54), and stimulates GnRH neurons to synthesize and secrete GnRH. GPR54 is a G protein-coupled receptor with seven transmembrane helices (29). This system is activated at the start of puberty and is the main trigger of pubertal onset and the regulator of GnRH neuron function in men (30).

In puberty, the levels of FSH, LH, testosterone, inhibin B, INSL3, growth hormone, and insulin-like growth factor-I (IGF-I) markedly increase, while AMH and sex hormone-binding globulin (SHBG) levels decline (31, 32). LH is the first hormone that increases in puberty, followed by an increase in INSL3 and testosterone levels approximately 4 months after the increase of LH levels (33).

The major changes in male puberty are increased testosterone production and the onset of spermatogenesis. The first sign of male puberty is the enlargement of testicular size; it exceeds 3 mL, as measured using an orchidometer. This occurs as a result of the onset of spermatogenesis and the growth of seminiferous tubules at the start of puberty (27). Testosterone, in association with FSH, stimulates spermatogenesis. Since sperm production markedly increases during pubertal progression, the testis enlarges dramatically during puberty and finally stops growing at the time of full pubertal maturation (34). Spermarche is the first appearance of mature spermatozoa in urine detected by laboratory analysis, and ejacularche is the first conscious ejaculation, which usually occurs approximately 1 year after testicular enlargement. Subsequently, testosterone induces male physical changes, including facial, body, and pubic hair growth; penile growth; increased libido; spontaneous penile erection; rapid growth rate (peak height velocity); increased muscle mass; decreased body fat; and finally, deepening of voice (27). The rapid increase in height during puberty results from an increased growth hormone, IGF-I, together with an increased testosterone secretion (35, 36).

## Pubertal development in boys with a history of cryptorchidism

### Physical changes during puberty

#### Age at onset of puberty in boys with a history of congenital cryptorchidism

Four studies investigated the age at the onset of puberty in boys with a history of cryptorchidism. A longitudinal follow-up study in 106 cryptorchid boys by Dickermann et al. showed that the onset of puberty based on the initiation of pubic hair growth was within the normal range of the non-cryptorchid boys (37). Taskinen et al. studied 76 men with a history of cryptorchidism with hormonal and/or surgical treatment between the ages of 10 months and 13 years and 47 healthy men in a cohort study in Helsinki, Finland. The age of the participants during the study was 16–30 years. The self-reported onset of first conscious ejaculation in men with a history of surgically or hormonally treated unilateral or bilateral cryptorchidism was significantly later than in men without cryptorchidism (14.0 *vs.* 13.0 years, respectively) (38). Arendt et al. studied 196 boys with a history of cryptorchidism and 7,502 non-cryptorchid boys in the Danish puberty cohort and found that self-reported onset of pubertal development, i.e., Tanner stage of genitalia, pubic hair, axillary hair, acne, voice break, and first ejaculation, was similar between the two groups regardless of the severity of cryptorchidism, birthweight, and maternal body mass index (BMI) (39). These three studies did not report the age when testicular size exceeded 3 mL, which is the first sign of puberty. Pubic hair growth (pubarche) can first appear because of an increased adrenal androgen secretion during adrenarche, even though prominent pubic hair growth is seen during puberty as a result of a markedly increased testosterone secretion. In addition, pubertal evaluation by a boy himself or his parents is not correlated well with clinical examination (40); therefore, this is a limitation of the assessment of pubertal onset. To date, one study reported the onset of puberty assessed by testicular size in boys with a history of cryptorchidism. Sadov et al. studied 46 boys with a history of congenital cryptorchidism and 65 non-cryptorchid boys in the Finnish birth cohort study. The boys were examined bi-annually from the age of 8.5 years to full pubertal maturation defined as the time when testicular size was stable for three consecutive visits. The age at onset of puberty (mean ± SD) defined as testicular size exceeding 3 mL by an orchidometer was similar between the two groups (11.7 ± 1.1 years vs. 11.8 ± 1.0 years, respectively) (41). In hypogonadotropic hypogonadism, cryptorchidism can be a presenting sign at birth and later causes the absence of puberty unless properly treated. However, hypogonadotropic hypogonadism is a very rare condition as compared to congenital cryptorchidism.

The height and penile length of the boys with a history of cryptorchidism were within the reference range of the non-cryptorchid boys (37, 41). The Finnish birth cohort study demonstrated that height at prepuberty, at the onset of puberty, and full pubertal maturation were similar between boys with and without a history of cryptorchidism. The between-group IGF-I and insulin-like growth factor-binding protein-3 (IGFBP-3) levels were generally similar from prepuberty to the end of puberty. This indicates that the growth axis is not affected by a history of cryptorchidism (42).

In conclusion, the age at the onset of puberty and other pubertal signs of boys with a history of congenital cryptorchidism do not differ from those of non-cryptorchid boys. However, the age at first conscious ejaculation in boys with a history of cryptorchidism is similar or occurs at a later age compared with that of boys without a history of cryptorchidism, showing the variability between studies, and further research might be needed to fully understand these relationships.

## Evaluation of testicular size during puberty

Testicular size is reported as testicular length assessed using a ruler or reported as testicular volume assessed using an orchidometer or testicular volume based on measurements by testicular ultrasonography. Ultrasonography-based testicular volume is calculated using one of the three most commonly used formulas: prolate spheroid formula (length × width^2^ × 0.52) (43), ellipsoid formula (length × width × height × 0.52) (44), or Lambert’s formula (length × width × height × 0.71) (45). These three methods have a strong correlation with actual testicular volume measured by water displacement (46). Testicular size measured using an orchidometer is generally larger than the one obtained by ultrasound because measurement by an orchidometer usually includes the epididymis and the scrotal skin (47). For the indicator of the onset of puberty, a testicular size of >3 mL by an orchidometer has a good correlation with a testicular length of >25 mm by a ruler (41). The testicular length of 25 mm by a ruler and testicular volume of 3 mL by an orchidometer correspond to the ultrasonography-based volumes of 1.7 mL and 1.6 mL (Lambert’s formula), respectively (41).

In addition to the identification of reduced-size testis, ultrasonography may also identify inhomogeneous parenchyma and microlithiasis of former cryptorchid testis (48).

## Pubertal testicular growth in healthy boys

From birth to before puberty, Sertoli cells are the main component of the testis, and because they are stimulated by FSH, testicular size is rapidly increased during minipuberty (20, 21). The testes slightly increase in size in childhood (49, 50). During the early phase of puberty, there is increased Sertoli cell proliferation, and seminiferous tubules increase in length, resulting in an increased testicular size (51). This is followed by the rapid and more marked testicular growth caused by an increased diameter of the seminiferous tubule owing to the progress of spermatogenesis (34, 51). This is the time when there is a shift of the main cellular component of the testis from Sertoli cells in prepuberty to germ cells in adulthood. Because of this, the adult testicular size is positively correlated with sperm concentration and total sperm count (44, 52).

## Pubertal testicular growth in congenital cryptorchidism

In the Finnish birth cohort study, the testicular size was measured every 6 months from prepuberty to the end of puberty (41). The modeled prepubertal volumes of the former cryptorchid testes (among boys with a history of unilateral or bilateral cryptorchidism) and those of the testes of non-cryptorchid boys were similar. However, the modeled final volumes of the former undescended testes (among boys with a history of unilateral or bilateral cryptorchidism) were smaller than the modeled final testicular volumes of boys without a history of cryptorchidism. Interestingly, the modeled final volumes of the descended testes of the boys with a history of unilateral cryptorchidism were larger than those of the testes of the non-cryptorchid boys. This suggests a compensatory increase in the size of the descended testis in unilateral cryptorchidism. The age at peak testicular growth was similar between cryptorchid and non-cryptorchid boys (41).

Compensatory testicular hypertrophy in unilateral cryptorchidism was also observed in the previous studies (53, 54). The overall prevalence of this condition in 1,257 unilaterally cryptorchid boys from pubic hair Tanner stage 1 to 5 in Israel was 12%. Compensatory testicular hypertrophy was noted since prepuberty in 40% of boys, at early puberty in 30%, in 16.7% at pubic hair Tanner stage 3, and in 13.3% after pubic hair Tanner stage 4 (53).

**Figure 1:**
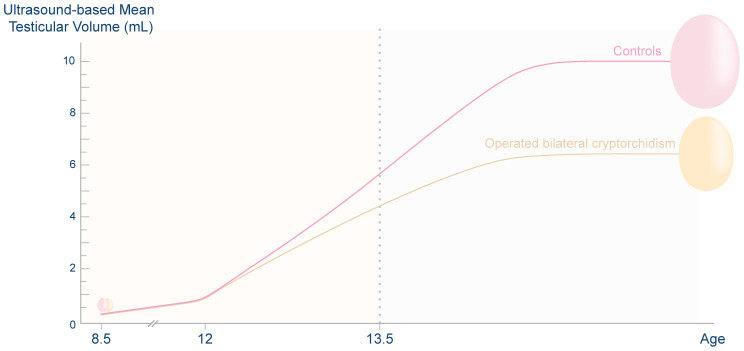
Ultrasound-based mean testicular volume during puberty of boys with a history of bilateral cryptorchidism who underwent orchiopexy in childhood and that of non-cryptorchid controls. During prepuberty, the mean testicular volume of the two groups of boys was similar. After pubertal onset, at the mean time of 11.8 years, the testes of both groups grew rapidly. However, the former group had smaller testes than controls from 1 year after pubertal onset to the end of puberty. Half of the testicular growth occurred by the age of approximately 13.4 years. Data were derived from Rodprasert et al. and Sadov et al. (41, 42) This figure is a simplified illustration of testicular growth in the two groups of boys; it is not for use as a reference of the testicular size of the population. The graph generated from the original data was presented in Rodprasert et al. (42).

When the combined testicular volume of both testes and the previous orchiopexy was considered, the combined testicular volume of boys with a history of operated unilateral or bilateral cryptorchidism was smaller than that of controls from approximately 1 year after pubertal onset to full pubertal maturation (42). **Figure 1** illustrates pubertal testicular growth in boys with a history of surgically treated bilateral cryptorchidism and controls. However, the combined testicular volume of the boys who had a history of spontaneously resolved unilateral or bilateral cryptorchidism did not differ from that of non-cryptorchid boys (41, 42). These findings are possibly due to a higher severity of testicular pathology in those forms of cryptorchidism that needed orchiopexy than in spontaneously resolved cryptorchidism. This study compared patterns of testicular growth and hormone levels of boys with a history of spontaneously resolved cryptorchidism to those of boys with a history of operated cryptorchidism. This is different from other previous studies in which these two groups were combined.

## Hormonal changes in minipuberty

A birth cohort study in Finland and Denmark showed higher FSH and lower to similar inhibin B levels in 3-month-old cryptorchid boys, indicating an impaired Sertoli cell function compared with non-cryptorchid controls (20). By contrast, serum FSH and inhibin B levels of cryptorchid and non-cryptorchid boys were similar in the studies in the Netherlands and the USA, suggesting preserved Sertoli cell function (55, 56). AMH levels were not different between boys with unilateral or bilateral cryptorchidism and non-cryptorchid controls between 1 and 6 months of age (55, 57).

Serum LH and testosterone levels in cryptorchid and non-cryptorchid boys were not different in some studies, suggesting normal Leydig cell function of cryptorchid boys during minipuberty (19, 20, 56, 58). Serum INSL3 levels, another marker of Leydig cell function, were measured in the birth cohort study in Finland and Denmark, which were also similar in cryptorchid and non-cryptorchid boys; however, the serum LH-to-INSL3 ratio was higher in the persistent cryptorchid boys at 3 months in Finnish and Danish boys, and the serum LH-to-testosterone ratio was higher in persistently cryptorchid boys than non-cryptorchid boys in Finland, suggesting some degree of decreased Leydig cell function (19). Another study showed higher LH but similar testosterone levels, suggesting compensated Leydig cell dysfunction in cryptorchid as compared with non-cryptorchid boys (20). In contrast, cryptorchid boys had lower plasma testosterone levels than non-cryptorchid boys in the older study; however, LH was not measured (59). Boys who had non-palpable testis or the testis was in the suprascrotal or inguinal position had lower serum testosterone levels than boys who had testis in the scrotal or high scrotal position (60).

Some studies compared the minipubertal hormone levels between cryptorchid boys who had spontaneous testicular descent during minipuberty and boys who had persistent cryptorchidism for longer than 3 months to 1 year (58, 61, 62). Boys with persistent cryptorchidism had lower LH and testosterone levels than boys who had spontaneous testicular descent in some studies (61–63). In contrast, basal and peak LH-releasing hormone (LHRH)-stimulated LH and basal and post-human chorionic gonadotropin (hCG)-stimulated serum testosterone levels of boys with persistent cryptorchidism, spontaneous testicular descent, and controls were generally similar in another study (58). The FSH levels were not different between the boys with persistent or transient cryptorchidism and controls (58, 61, 62).

In summary, in cryptorchid boys, the Sertoli cell’s hormone secretion is normal or reduced, whereas Leydig cell function seems to be preserved or mildly affected during minipuberty. Hormonal data of boys with spontaneous testicular descent and persistent cryptorchidism vary across studies.

## Hormonal changes in puberty

### FSH–inhibin B axis

A longitudinal study followed 106 boys with a history of congenital cryptorchidism starting from the age of 5 to 14 years to full pubertal maturation and compared hormone levels with normal reference ranges (37). This study showed elevated basal and GnRH-stimulated plasma FSH levels in boys with a history of cryptorchidism as compared with the normal reference range, indicating a primary testicular defect affecting Sertoli cells and/or germ cells. The Finnish birth cohort study additionally investigated the effects of orchiopexy and spontaneous testicular descent on hormone levels (42). The study followed 30 boys with a history of unilateral cryptorchidism and 16 boys with a history of bilateral cryptorchidism who had experienced spontaneous testicular descent or underwent orchiopexy and 63 non-cryptorchid boys from the age of 8.5 years to the end of puberty. The mean age at the first orchiopexy was 2.0 years (range, 0.26–4.3 years). Higher FSH and comparable inhibin B levels in boys with previous unilateral cryptorchidism with either spontaneous testicular descent or orchiopexy compared with non-cryptorchid boys were found from 2 years after the onset of puberty to the end of puberty, which suggested compensated Sertoli cell and/or germ cell dysfunction and/or decreased number of these cells in unilateral cryptorchidism. Considering the normal testicular volume in boys with a history of unilateral cryptorchidism and spontaneous testicular descent, the number of Sertoli cells and germ cells is probably preserved, but the cell function is impaired, in these boys. In boys with a history of bilateral cryptorchidism, particularly in those who underwent orchiopexy, higher FSH and lower inhibin B levels than those of non-cryptorchid boys from 0.5–1 year after pubertal onset to the time of full pubertal maturation were shown, indicating decompensated Sertoli cell and/or germ cell dysfunction and/or more severely decreased number of these cells in bilateral cryptorchidism (42). In another study, boys with a history of unilateral cryptorchidism and compensatory hypertrophy of the contralateral descended testis had higher basal and LHRH-stimulated FSH levels than normal boys from prepuberty to the end of puberty (53). The findings confirm the previous study that cryptorchid boys have primary testicular damage rather than the pathology in the hypothalamus or pituitary gland. It can also be implied that boys who needed orchiopexy might have more severe testicular pathology than boys who had spontaneous testicular descent. In contrast, a cross-sectional study in 1977 in 154 cryptorchid boys, aged 1 month to 15 years, reported similar basal and LHRH-stimulated FSH levels in boys with a history of bilateral or unilateral cryptorchidism and controls (62). This result suggested a preserved Sertoli cell function in cryptorchidism.

### LH–testosterone axis

A study in 1979 showed elevated basal and GnRH-stimulated plasma LH levels and decreased basal and hCG-stimulated testosterone levels in previously cryptorchid boys, indicating a primary Leydig cell defect (37). By contrast, the Finnish birth cohort study found preserved Leydig cell function shown by similar LH, testosterone, and INSL3 in previously unilaterally or bilaterally cryptorchid boys with either spontaneous testicular descent or orchiopexy as compared with non-cryptorchid boys (42). These findings are consistent with the pubertal assessment in some studies, which showed similar male physical changes in boys with and without a history of cryptorchidism (37, 39, 41). In contrast, a cross-sectional study in 1977 found lower basal and LHRH-stimulated LH levels and hCG-stimulated testosterone in cryptorchid boys than controls from the age of 2 years to pubertal stage P2, suggesting impaired pituitary LH secretion and Leydig cell testosterone secretion in cryptorchid boys in prepuberty until early puberty (62, 63). After pubertal stage P2, these differences disappeared (62, 63).

**Figure 2:**
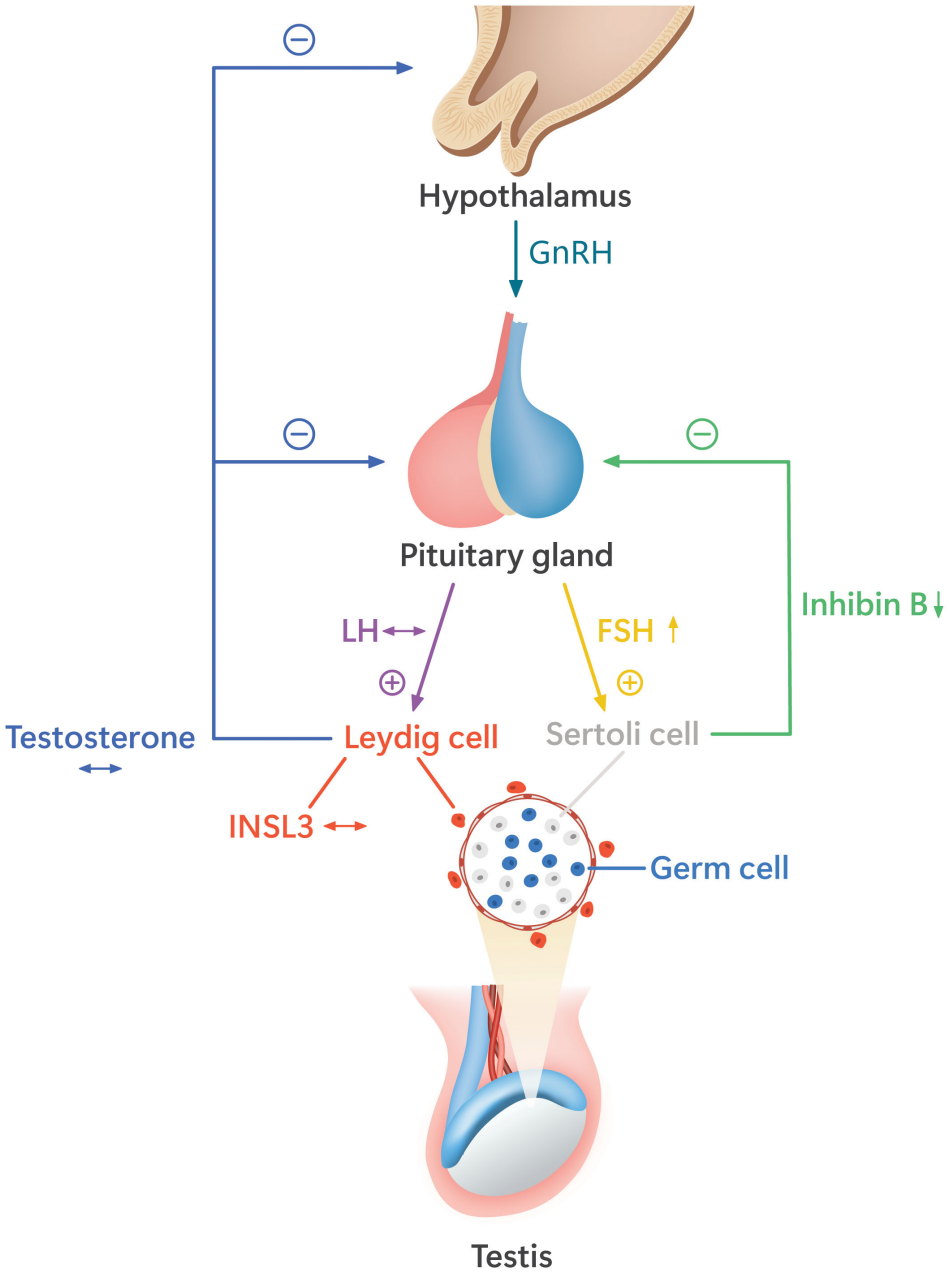
The alterations of the hypothalamic–pituitary–gonadal (HPG) axis function in pubertal boys with a history of bilateral cryptorchidism who underwent orchiopexy before puberty. These boys have higher follicle-stimulating hormone (FSH) and lower inhibin B than non-cryptorchid boys, indicating decreased function and/or number of Sertoli cells and germ cells. The levels of luteinizing hormone (LH) and the Leydig cell hormones, testosterone, and insulin-like peptide 3 (INSL3) are similar to those of non-cryptorchid controls (42). The hormone levels as compared with the normal range are indicated by arrow signs: ↔, the levels similar to the normal range; ↑, the levels higher than the normal range; ↓, the levels lower than the normal range.

**Figure 2** illustrates the alterations of HPG axis hormones in boys with a history of bilateral cryptorchidism who underwent orchiopexy in childhood (42).

In conclusion, the results of the Leydig cell function varied across studies. However, the latest longitudinal study in Finland, which used newer assays for hormone measurements, showed preserved Leydig cell function in cryptorchidism. The older studies used insensitive radioimmunoassays for the measurement of gonadotropin and testosterone levels. The low sensitivity of these assays may have caused poor detection of low FSH, LH, and testosterone levels, particularly during early puberty. In addition, inhibin B levels were not measured; therefore, the evaluation of Sertoli cell and germ cell function was based solely on FSH.

Adult men with a history of bilateral cryptorchidism had lower inhibin B and higher FSH and LH levels but similar testosterone levels as compared with non-cryptorchid controls (64, 65), whereas men with a history of unilateral cryptorchidism had similar FSH, LH, testosterone, and low inhibin B levels as compared with non-cryptorchid controls (65, 66). Testosterone level further declines with increasing age at orchiopexy (67). The findings in adulthood are rather consistent and showed the continuous decrease of Sertoli cell and germ cell function and/or number but relatively preserved Leydig cell function. Hormonal changes in congenital cryptorchidism from birth to adulthood were also described in our previous review (11).

### IGF-I levels in congenital cryptorchidism

The Finnish birth cohort study showed that IGF-I and IGFBP-3 levels in boys with a history of cryptorchidism did not differ from those in non-cryptorchid boys (42). This result supports the finding in the same and other studies that the height of the boys in the two groups was similar from prepuberty to adulthood (37, 41, 42, 68).

## Histopathology of congenital cryptorchidism

The number of germ cells in the testes of patients with cryptorchidism during the first year of life is similar to those in non-cryptorchid controls. The germ cell number decreased remarkably from 1 to 3 years of age (69). The number of germ cells and Sertoli cells in the cryptorchid testis was lower in boys who underwent orchiopexy at the age of 3 years compared with orchiopexy at the age of 9 months, indicating more severe germ cell and Sertoli cell loss with delayed operation (70). This was observed in both unilateral and bilateral cryptorchidism (70). Another study also showed similar findings (71). The transformation from gonocytes into Ad spermatogonia was reported to be impaired, which explained the finding of a reduced number of Ad spermatogonia per seminiferous tubule cross-section in the first year of life of cryptorchid boys (72, 73). In addition, the maturation of Ad spermatogonia into primary spermatocytes was defective (73). Fibrosis was also detected in undescended testes (74).

Studies on testicular pathology of congenital cryptorchidism during puberty are scarce because most boys undergo orchiopexy in childhood. A study on simultaneous testicular biopsy and orchiopexy in unilaterally cryptorchid boys between the ages of 10 and 12 years showed that the number of spermatogonia per tubular cross-section of these boys was lower than normal (75). Some testes showed Sertoli cells only or the number of spermatogonia per tubular cross-section of less than 1% (75, 76). A study including 187 boys with a history of cryptorchidism aged 1 to 209 months showed germ cell depletion and Leydig cell loss, which worsened with each month of increasing age at orchiopexy (71).

Decreased germ cell number and Sertoli cell-only syndrome can explain high FSH and low inhibin B levels in pubertal boys and low sperm count in adult men (65, 77). Studies on Leydig cell number during puberty to adulthood varied from Leydig cell depletion (71), preserved Leydig cell number (78), and Leydig cell hyperplasia (79, 80). These pathological findings might explain the mixed results of Leydig cell function as discussed earlier.

## Effects of spontaneous testicular descent and orchiopexy on testicular growth from birth to puberty in congenital cryptorchidism

In a longitudinal study of 91 boys with congenital unilateral cryptorchidism in Sweden, boys were followed from birth to the age of 5 years, and 82% of boys showed spontaneous testicular descent before the age of 2 months (81). However, 22% of these spontaneously descended testes went back to the cryptorchid position; i.e., the boys had recurrent cryptorchidism. The testicular volumes of the three groups of boys were compared: boys with spontaneously descended testis, boys with orchiopexy at 9 months (early orchiopexy group), and boys with orchiopexy at 3 years (late orchiopexy group). At birth, the mean testicular volumes of the cryptorchid testes between the three groups were similar. During the entire study period, the normally descended testis was larger than the cryptorchid testis and previously cryptorchid testis with spontaneous descent. However, from the age of 2 years onward, the cryptorchid testes of the late orchiopexy group were smaller than those of the other two groups. The spontaneously descended testes were significantly larger than the testes of the late orchiopexy group at the ages of 2, 4, and 5 years. The boys with spontaneous testicular descent had a significantly higher mean ratio between the cryptorchid and scrotal testes, indicating a better growth of the cryptorchid testes than the growth of the late orchiopexy group at the ages of 6 months and 1, 2, 4, and 5 years (81). The early orchiopexy group showed significant testicular growth at the ages of 2, 3, and 4 years as compared with at the age of 6 months. On the contrary, the late orchiopexy group did not show significant testicular growth (82). Similarly, another study showed that early orchiopexy before 2 years of age is associated with better catch-up testicular growth in unilateral cryptorchidism (83). These results demonstrated that in terms of testicular growth in childhood, the spontaneously descended testis has better testicular growth than the testis that needs orchiopexy, and early surgical treatment is more beneficial than late operation in congenital cryptorchidism. The Nordic consensus in 2006 recommends orchiopexy between 6 and 12 months of age or at the time of diagnosis if the diagnosis is made later than 12 months (84). The American Urological Association (AUA) guideline in 2014 recommends orchiopexy in the first 18 months of age (85). Finally, the updated European Association of Urology (EAU) management guidelines for cryptorchidism recommend orchiopexy before the age of 12 months and by 18 months at the latest (84–86).

During puberty, the Finnish birth cohort study specifically investigated the effects of laterality and management of congenital cryptorchidism on testicular volume and reproductive hormone levels (42). The mean age at the first orchiopexy was 2.0 years (range, 0.26–4.3 years). This study demonstrated that the combined testicular volumes of boys with operated unilateral or bilateral cryptorchidism were significantly smaller than those of non-cryptorchid boys from approximately 1 year after pubertal onset to the end of puberty, while the size of the spontaneously descended testes did not differ from that of non-cryptorchid testes during the entire pubertal period. The hormonal data, as mentioned above, showed higher FSH and similar inhibin B levels in boys with a history of operated unilateral cryptorchidism compared with non-cryptorchid boys from 2 years after the onset of puberty to the end of puberty, which suggested compensated Sertoli cell and/or germ cell function and/or decreased number of these cells. In boys with a history of operated bilateral cryptorchidism, higher FSH and lower inhibin B levels than in the non-cryptorchid boys from 0.5–1 year after pubertal onset to the time of full pubertal maturation were shown, indicating decompensated Sertoli cell and/or germ cell function and/or more severely decreased number of these cells. Leydig cell function did not differ between the groups. This study showed that both laterality and management of cryptorchidism influence testicular growth and function during puberty. Bilateral cryptorchidism seems to have more severe testicular damage than unilateral cryptorchidism, even though direct comparisons between these two entities were not performed because of the reduced statistical power with a limited number of participants. Cryptorchidism that required orchiopexy could not catch up with testicular growth as compared with descended testis even after the operation and had more severe damage to Sertoli cells and/or germ cells than spontaneously descended testis. This study did not examine the long-term effects of early and late orchiopexy during puberty (41, 42). These results are consistent with the above-mentioned studies on childhood.

In adult men with a history of cryptorchidism, the operated testes were smaller than the spontaneously descended testes. In men with a history of unilateral cryptorchidism, the operated testis was also smaller than the contralateral normally descended testis (65, 87). These results support the findings in puberty. One study did not find contralateral testicular hypertrophy in men with a history of unilateral cryptorchidism when compared with controls (65), which is different from a study in pubertal boys (41).

## Acquired cryptorchidism and puberty

Studies on mechanisms of acquired cryptorchidism are scarce and showed mixed results. In one series, approximately half of the cases were associated with a small opening of processus vaginalis and normal attachment of the gubernaculum (88). A study by Alchoikani et al. (2021) found an abnormal attachment of the gubernaculum to the proximal part of the junction between the upper lateral part of the scrotum and the medial thigh. All cases showed patent processus vaginalis, and the length of patent processus vaginalis varied. The separation between the epididymal head and the upper pole of the testis was detected, and also the hydatid of Morgagni was observed (89). A Dutch study found that compared to cases with congenital cryptorchidism, cases with acquired cryptorchidism are more likely to have normal insertion of the gubernaculum and a closed processus vaginalis (90). It has been suggested that defects in the elongation of spermatic cord structures resulting from an incomplete disappearance of processus vaginalis may play a role in acquired cryptorchidism (4).

Data on the testicular growth and function in individuals with a history of acquired cryptorchidism, specifically during puberty, are lacking. However, some studies reported the combined pubertal and adult data. Spontaneous testicular descent at the time of puberty occurred in 57.0%–77.5% of the acquired cryptorchid testis—most of which occurs in early puberty to midpuberty (mean age, 12.9 years) (91, 92). However, 22.5%–43.0% remained cryptorchid and needed orchiopexy to correct cryptorchidism at puberty. Among boys and men with a history of unilateral cryptorchidism, there were non-significantly higher percentages of the testes that were smaller than the contralateral, descended testes of more than 1 mL in the orchiopexy at puberty group *vs.* spontaneous testicular descent group (91). A higher chance of spontaneous testicular descent is associated with advancing age (91). The author of this article concluded that a wait-and-see approach in which orchiopexy is performed at puberty in boys who did not have spontaneous testicular descent does not cause a negative impact on testicular growth (91).

One study observed testicular growth of acquired cryptorchidism following prepubertal orchiopexy in 105 boys and men aged 14.0–31.6 years (mean 25.7 years) (93). The age at orchiopexy ranged from 2.4 to 13.9 years (mean, 9.2 years). This study showed that almost all testes (99.3%) were in a low scrotal position during the study participation. The size of the operated testis of unilateral cryptorchidism was similar to that of the operated testis of bilateral cryptorchidism but was significantly smaller than that of the contralateral normally descended testis and the normative data of testicular size in the general population (93). This finding agrees with the data on congenital cryptorchidism. The age at orchiopexy was not correlated with testicular volume during follow-up (93).

One study compared testicular growth of acquired undescended testes that had spontaneous descent or orchiopexy at puberty (92). The mean age of spontaneous descent was 12.9 years (ranging from 9.8 to 16.9 years), while the mean age at pubertal orchiopexy was 14.3 years (ranging from 11.7 to 18 years). The testicular growth during a mean follow-up of 4.7 years (ranging from 0.1 to 12.0 years) was generally similar between the two groups, suggesting that a wait-and-see approach does not result in impaired testicular growth (92).

To our knowledge, data on pubertal reproductive hormone levels in acquired cryptorchidism compared with reference levels or hormone levels of non-cryptorchid boys are currently not available. However, a Dutch study compared the reproductive hormone levels between the previously acquired cryptorchid men operated at diagnosis (mean age at orchiopexy of 10.7 years) and men who received wait-and-see protocol, in which spontaneous descent was waited until Tanner stage P2G2 and orchiopexy was performed in the case of non-descent (mean age at orchiopexy of 13.2 years) (94). The men were between 18.0 and 35.8 years of age at the time of the study. Testicular volume, serum FSH, LH, testosterone, and inhibin B levels in the two groups were similar in men with a history of either unilateral or bilateral acquired cryptorchidism with either of the treatment protocols, except that inhibin B levels of men with a history of unilateral acquired cryptorchidism and orchiopexy at diagnosis were significantly lower than those of men who received wait-and-see protocol. Men with a history of acquired bilateral cryptorchidism who underwent orchiopexy after 10 years of age had larger testis than men who had orchiopexy before the age of 10 years (94). In men with a history of acquired unilateral cryptorchidism, the descended testes were smaller than the contralateral normally descended testes (48)—the finding is similar to that of congenital cryptorchidism (41). However, one study showed that the contralateral normally descended testes were smaller than those of non-cryptorchid controls (48). This result is different from a study in congenital cryptorchidism, which demonstrated that the contralateral normally descended testes were larger than those of non-cryptorchid controls (41). The testicular histopathology of acquired cryptorchidism is similar to that of congenital cryptorchidism (95–97).

So far, the management of acquired cryptorchidism is still unclear. Some recommend orchiopexy before puberty, but some recommend waiting until puberty because almost 80% have spontaneous descent at puberty, particularly if cryptorchid testis stays in the superficial inguinal pouch (92, 98), and perform orchiopexy only when spontaneous descent does not occur.

## Conclusion

The currently available data show that previously congenital cryptorchid boys generally have normal physical development during puberty, including normal age at the onset of puberty indicated by testicular enlargement, penile length, pubic hair, axillary hair, acne, voice break, and height. However, there is evidence suggesting a potential delay in the age of first conscious ejaculation. In addition, testicular growth is impaired in unilateral and bilateral cryptorchidism requiring orchiopexy in childhood. Boys with a history of cryptorchidism, particularly bilateral cryptorchidism with orchiopexy, have an increased FSH and decreased inhibin B levels, suggesting an impaired Sertoli cell/germ cell function and/or decreased number of these cells during puberty, which can result in poor spermatogenesis in adulthood. Testosterone and LH levels measured using new assays in boys with and without a history of cryptorchidism did not differ, indicating a generally preserved Leydig cell function and/or number.

Data on pubertal boys with a history of acquired cryptorchidism are scarce. However, impaired testicular growth seems similar to congenital cryptorchidism—those who needed orchiopexy have poorer testicular growth during puberty. More data on pubertal hormone levels in boys with acquired cryptorchidism are needed.

## Author contributions

WR: Conceptualization, Writing – original draft, Writing – review & editing. HV: Conceptualization, Supervision, Writing – original draft, Writing – review & editing. JT: Conceptualization, Supervision, Writing – original draft, Writing – review & editing.
